# Symbiotic bacteria Sodalis glossinidius, Spiroplasma sp and Wolbachia do not favour Trypanosoma grayi coexistence in wild population of tsetse flies collected in Bobo-Dioulasso, Burkina Faso

**DOI:** 10.21203/rs.3.rs-4756528/v1

**Published:** 2024-08-28

**Authors:** Youssouf Mouliom Mfopit, Etienne Bilgo, Soudah Boma, Martin Bienvenu Somda, Jacques Edounou Gnambani, Maurice Konkobo, Abdoulaye Diabate, Guiguigbaza-Kossigan Dayo, Mohammed Mamman, Soerge Kelm, Emmanuel Oluwadare Balogun, Mohammed Nasir Shuaibu, Junaidu Kabir

**Affiliations:** Institute of Agricultural Research for Development (IRAD); Institut de Recherche en Sciences de la Santé; Centre international de recherche-développement sur l’elevage en zone subhumide; Centre international de recherche-développement sur l’elevage en zone subhumide; Institut de Recherche en Sciences de la Santé; Institut de Recherche en Sciences de la Santé; Institut de Recherche en Sciences de la Santé; Centre international de recherche-développement sur l’elevage en zone subhumide; Ahmadu Bello University; University of Bremen; Ahmadu Bello University; Ahmadu Bello University; Ahmadu Bello University

**Keywords:** Tsetse flies, Wolbachia, Spiroplasma, Sodalis glossinidius, Trypanosoma grayi, Burkina Faso

## Abstract

**Background.:**

Tsetse flies, the biological vectors of African trypanosomes, have established symbiotic associations with different bacteria. Their vector competence is suggested to be affected by bacterial endosymbionts. The current study provided the prevalence of three tsetse symbiotic bacteria and trypanosomes in *Glossina* species from Burkina Faso.

**Results.:**

A total of 430 tsetse flies were captured using biconical traps in four different collection sites around Bobo-Dioulasso (Bama, Bana, Nasso, and Peni), and their guts were removed. Two hundred tsetse were randomly selected and their guts were screened byPCR for the presence of *Sodalis glossinidius, Spiroplasmasp*., *Wolbachia* and trypanosomes. Of the 200 tsetse, 196 (98.0%) were *Glossina palpalis gambiense*and 4 (2.0%) *Glossina tachinoides*. The overall symbiont prevalence was 49.0%, 96.5%, and 45.0%, respectively for *S. glossinidius, Spiroplasma* and *Wolbachia*. Prevalence varied between sampling locations: *S. glossinidius*(54.7%, 38.5%, 31.6%, 70.8%); *Spiroplasma* (100%, 100%, 87.7%, 100%); and *Wolbachia*(43.4%, 38.5%, 38.6%, 70.8%),respectively in Bama, Bana, Nasso and Peni. Noteworthy, no *G. tachhnoides*was infected by *S*. *glossinidius* and *Wolbachia*, but they were all infected by *Spiroplasma sp*. A total of 196 (98.0 %) harbored at least one endosymbionts. Fifty-five (27.5%) carried single endosymbiont. Trypanosomes were found only in *G.p. gambiense*, but not *G. tachinoides*. Trypanosomes were present in flies from all study locations with an overall prevalence of 29.5%. In Bama, Bana, Nasso, and Peni, the trypanosome infection rate was respectively 39.6%, 23.1%, 8.8%, and 37.5%. Remarkably, only *Trypanosoma grayi* was present. Of all trypanosome-infected flies, 55.9%, 98.3%, and 33.9% hosted *S. glossinidius, Spiroplasma sp* and *Wolbachia*, respectively. There was no association between *Sodalis*, *Spiroplasma* and trypanosome presence, but there was a negative association with *Wolbachia* presence. We reported1.9 times likelihood of trypanosome absence when *Wolbachia* was present.

**Conclusion:**

This is the first survey reporting the presence of *Trypanosoma grayi* in tsetse from Burkina Faso. Tsetse from these localities were highly positive for symbiotic bacteria, more predominantly with *Spiroplasma sp*. Modifications of symbiotic interactions may pave way for disease control.

## Background

Tsetse flies (*Glossina* spp.) are the only cyclic vectors of African trypanosomes, the causative agents of trypanosomiasis in both humans and animals. Trypanosomiasis is one of the major endemic diseases in sub-Saharan Africa. It is a threat of human health and considered as one of the major constraints to animal production contributing to poverty and food insecurity in Africa [1, 2]. Tsetse flies host diverse microorganisms including viruses, bacteria, and fungi. The bacteria are environmentally acquired or maternally transmitted. Several bacterial genera acquired from the environment have been detected in tsetse [3–6]. The microbiota composition varies depending both on tsetse species and the geographic origin of tsetse. Symbiotic associations with *Wigglesworthia glossinidia, Sodalis glossinidius, Wolbachia*, and *Spiroplasma* spp. have been described [7]. Symbionts influence tsetse physiology, including fecundity, immunity, and nutrition [8]. They are thought to modulate the vectorial competence of tsetse flies and may therefore have the potential for vector and disease control [7, 8].

*Wigglesworthia glossinidia* is an obligate mutualist bacteria found in all tsetse species. This primary symbiont provides dietary supplements that are necessary for host fecundity and the maturation process of the adult immune system [7]. Tsetse’s second and facultative endosymbiont is the commensal *S. glossinidius*. It has been suspected to play a role in potentiating susceptibility to trypanosome infection in tsetse by influencing the efficacy of the tsetse immune system [9] possibly through lectin-inhibitory activity. *Sodalis glossinidius* can be transmitted between tsetse flies trans-ovarially, vertically, and horizontally [7, 10]. The third symbiont, known as *Wolbachia*, is trans-ovarially transmitted between different generations of tsetse flies. *Wolbachia* manipulates the reproductive biology of its host through a variety of mechanisms, such as cytoplasmic incompatibility, parthenogenesis, male killing, and feminization [11], therefore impairing host fertility, lifespan, and immunity. Recently, *Spiroplasma* has been established as a fourth tsetse endosymbiont in some natural tsetse populations and laboratory colonies. The role of *Spiroplasma* in the tsetse fly host is currently unclear. However, reproductive alterations such as cytoplasmic incompatibility, male-killing, and sex determination are related to numerous species of *Spiroplasma* [3]. Conversely, some *Spiroplasma* strains might have a positive effect on their hosts, conferring resistance against pathogens. In tsetse flies, *Spiroplasma* was demonstrated to decrease vector competence [7, 12]. Therefore, *Spiroplasma* could be used to reduce trypanosome transmission in the tsetse fly.

In Burkina Faso, tsetse flies pose a significant threat to human health and livestock through the transmission of trypanosomes. The northern distribution limit of tsetse flies was decreased toward southern by a combination of climate change and anthropization [13], and 9 out of the 13 regions of the country are infested by tsetse flies [14]. Moreover, in the context of the progressive control pathway through the national atlas of tsetse and African animal trypanosomiasis (AAT), entomological data showed the presence of four tsetse species in Burkina Faso. The most widespread and abundant species are *Glossina tachinoides* (56.35%) and *G. palpalis gambiensis* (35.56%), and the lower densities of tsetse species are *G. morsitans submorsitans* (6.51%) and *G. medicorum* (less than 0.25%) [14].

The current control measures mainly rely on chemotherapies which are facing numerous challenges including the high financial burden for livestock owners, toxicity, and the development of drug resistance [15–17]. Significant efforts have been made in recent years to generate knowledge and develop strategies to control this disease within the country and throughout the West African region. Burkina Faso, through the Insectarium of the “*Centre International de Recherche-Développement sur l’Elevage en zone Subhumide*” (CIRDES) and the “Insectarium de Bobo Dioulasso-Campagne d’Eradication de la mouche Tsé-tsé et de la Trypanosomose” (IBD-CETT, formerly PATTEC Burkina Faso), produced a large quantity of sterile tsetse pupae for various control campaigns. Pupae were sent to Senegal to be used in the Sterile Insect Technique (SIT), and this collaboration allowed the eradication of the population of *G. p. gambiensis* from the Niayes and the transmission of animal trypanosomiasis has been interrupted in the treated area [18]. The use of the SIT, which can be complemented by symbiont-based approaches, is a promising option to increase the effectiveness of field interventions against trypanosomiasis.

Roles of facultative tsetse symbionts are not clearly understood and require further research. In Burkina Faso, few studies investigated the tripartite interactions between tsetse, trypanosomes and endosymbionts [19, 20]. Therefore, more data on tsetse symbionts need to be generated, for the implementation symbiont-based strategies which are key components for the complete interruption of trypanosome transmission. The study was therefore conducted to establish the prevalence of *S. glossinidius, Spiroplasma* and *Wolbachia* endosymbionts and their coexistence with *Trypanosoma* sp. in wild populations of tsetse species found around Bobo-Dioulasso, Burkina Faso, with the final goal of generating more data on the association tsetse-symbionts-trypanosomes.

## Methodology

### Study areas

**Figure 1:**
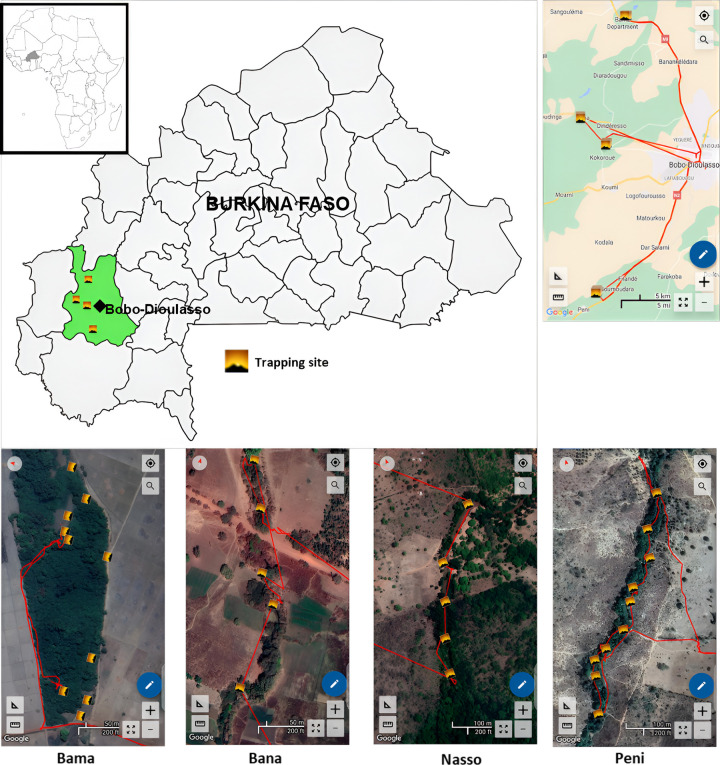
Map of study area. Trapping sites are marked with yellow squares. Tsetse flies were collected in Bama (10 traps), Bana (5 traps), Nasso (5 traps) and Peni (10 traps). (Map created with mapchart.net and SW Map).

Tsetse flies used in this study were captured in May 2023 in four peri-urban areas of Bobo-Dioulasso (Bama, Bana, Nasso, and Peni) located in the South-West of Burkina Faso (Fig. 1). The landscape that was originally savannah has now been replaced by crops (mainly cotton but also millet and maize). The climate is of the Sudano-Guinean type. The rainy season extends from May to September. The rainfall is around 1000 mm/year, with the rainiest month being August (281.51 mm). From 1992 to 2021, the average temperature was 27.55 °C with minima of 13.17 °C in December and maxima of 41.54 °C in April. The vegetation is composed of a mosaic of savannahs, and some forest galleries remain along the Kou Valley, an affluent of Mouhoun River, and constitute the main habitat of the riverine tsetse species *G. p. gambiensis*. During the dry season, sacred woods maintain permanent water and protected vegetation, as well as some monitor lizards (*Varanus niloticus*), crocodiles (*Crocodylus niloticus*), livestock, and humans that probably constitute the main feeding source of the tsetse fly population [21, 22].

### Tsetse trapping and dissection

Tsetse flies were captured in May 2023 with biconical traps [23]. In Bama and Peni, ten traps were deployed, while five traps were deployed in Bana and Nasso at approximately 200 meter intervals. Traps were deployed for 4 consecutive days, and flies were collected once a day and transported in cool boxes to the laboratory, where they were identified using morphological identification keys [24, 25].

The tsetse flies were dissected at the parasitology laboratory of the CIRDES (Bobo-Dioulasso, Burkina Faso). They were dissected in a drop of sterile saline solution [26]. The entire gut was isolated and preserved in a nucleic acid preservation agent (NAPA: 25 mM sodium citrate, 10 mM EDTA, 70 g ammonium sulphate/100 mL solution, pH 7.5) in 1.5 mL Eppendorf tubes and stored at −20°C for molecular analysis.

### DNA extraction

Molecular analysis was carried out at the molecular biology laboratory of the “Institut de Recherche en Sciences de la Santé” (IRSS, Bobo-Dioulasso, Burkina Faso). Gut samples were homogenised using a 2.0 mm metal bead on Tissue Lyser II (Qiagen, Hilden- Germany) for 30 s at a frequency (1/s) of 30. The genomic DNA extraction kit 2 (NIMR, Lagos, Nigeria) was used to extract DNA from the homogenates according to the manufacturer instructions. DNA yield and purity assessment were performed using Nanodrop Lite spectrophotometer (Thermo Scientific).

### Molecular detection of endosymbionts

The presence of symbionts was determined using symbiont species-specific PCR amplification assay as described by Mfopit et al. [27]. For detection of *Sodalis*, HemF (5’-ATGGGAAACAAACCATTAGCCA-3’) and HemR (5’-TCAAGTGACAAACAGATAAATC-3’) primers [28] were used to amplify the 650 bp fragment of the haemolysin gene. The presence of *Wolbachia* was detected by the amplification of a 438 bp fragment of the 16S rRNA gene with the primers WspecF (5’-CATACCTATTCGAAGGGATAG-3’) and WspcR (5’-AGCTTCGAGTGAAACCAATTC-3’) [29]. Screening for *Spiroplasma* was carried out by amplifying the 455 bp fragment of 16S rRNA gene with specific primers 63F (5’-GCCTAATACATGCAAGTCGAAC-3’) and TKSSsp (5’-TAGCCGTGGCTTTCTGGTAA-3’) as described by Doudoumis et al. [3].

The reactions were made of 20 μL containing 1× DreamTaq buffer, 150 μM dNTPs, 0.2 μM of each primer, 0.5 U of DreamTaq polymerase, and 100 ng of template DNA. Positive controls for each symbiont were used. For the negative control, nuclease-free water was used instead of fly DNA. The PCR conditions were as follows: 95°C for 5 min followed by 35 cycles at 95°C for 30 s, 54°C (*Sodalis* and *Wolbachia*), or 59°C (*Spiroplasma*) for 30 s, and 72°C for 30 s. The final elongation was at 72°C for 10 min.

Positive controls for *Wolbachia, Spiroplasma*, and *Sodalis* were positive samples from our previous study that were confirmed by sequencing [27].

To evaluate amplifications, 5 μL PCR reactions were analysed by electrophoresis on 1.5% agarose gel, which was stained with ethidium bromide and visualised using a transilluminator. Representative positive samples were re-amplified and sent for sequencing.

### Molecular detection of trypanosomes

Trypanosomes were detected by amplifying in a nested PCR, the trypanosome internal transcribed spacer (ITS1) region. For the nested PCR, two consecutive PCR reactions were carried out; a set of outer primers (TRYP-3: 5’-TGCAATTATTGGTCGCGC-3’ and TRYP-4: 5’-CTTTGCTGCGTTCTT-3’) were used in the first round reaction, followed by inner primers (TRYP-1: 5’-AAGCCAAGTCATCCATCG-3’ and TRYP-2: 5’-TAGAGGAAGCAAAAG-3’) in the second round as described by Adams et al. [30].

The first round of PCR was performed with modifications in a reaction mixture of 20 μL containing 1× of Firepol^®^ Master Mix (SOLIS BIODYNE), 0.2 μM of each primer, and 2 μL of template DNA. A *T grayi* positive control was used. For negative control, nuclease-free water was used instead of fly DNA. The PCR conditions were as follows: initial denaturation at 95°C for 3 min, followed by 30 cycles at 94°C for 1 min, 54°C for 30 s, and 72°C for 30 s, then a final elongation at 72°C for 5 min.

Thereafter, the first PCR products were diluted 20 fold and 1 μL was used as DNA template for the second PCR reaction with inner primers under the same conditions as described above.

Amplified products were subjected to electrophoresis on a 1.5% agarose gel stained with ethidium bromide and visualised using a transilluminator. Representative positive PCR products were re-amplified and sent for sequencing.

### Phylogenetic analysis

Obtained sequences were analysed using Geneious Pro version 5.5.9 software [31] and then subjected to BLAST search at the National Center for Biotechnology Information (NCBI, https://www.ncbi.nlm.nih.gov/) database to determine the closest related sequences in the GenBank.

Related gene sequences were aligned using the MUSCLE [32] alignment tool of MEGA X software [33] with its default setting. The software was also used to infer phylogenetic relationships. The maximum likelihood method was performed with the Kimura-2 model [34] for *Sodalis, Spiroplasma, Wolbachia* and trypanosome, as determined by the MEGA model finder tool with 1000 bootstrap replicates.

### Statistical analysis

Data were analysed using Microsoft Excel for Windows and IBM SPSS statistics version 20. Pearson’s chi-square analysis was employed to compare prevalence rates. A binary logistic regression was used to assess the association between symbiotic bacteria and the trypanosome infection. For expected values under 5, Fisher’s exact test was used. The statistical significance (*p* < 0.05) at a 95% confidence interval was considered.

## Results

### Entomological survey

During four days of trapping, a total of 430 tsetse were captured in the four collection sites around Bobo-Dioulasso (Bama, Bana, Nasso, and Peni). They belonged to two morphologically distinct species: 426 *G*. *p. gambiense* (99.1%) and four *G. tachinoides* (0.9%). The four *G. tachinoides* were all from Nasso. Most flies were captured in Bama (320), followed by Nasso, Peni, and Bana with respectively 73, 24, and 13 flies (Table 1). Considering the number of trapping days and number of traps used in each site, the general apparent density was 3.5 flies per trap per day (F/T/D) and was respectively 10.7, 7.3, 1.3, and 1.2 F/T/D in Bama, Nasso, Bana, and Peni (Table 1).

From the captured flies, 250 were dissected, and 200 gut samples were used for DNA extraction and subsequent molecular analysis: 196 *G. p. gambiense* (98.0%), and all four *G. tachinoides* (2.0%). Male flies were 95 (47.5%), while females were 105 (52.5%).

### Occurrence of Sodalis glossinidius in tsetse flies

The presence of *S. glossinidius* was investigated in 200 tsetse guts using a *Sodalis Hemolysin* gene-based PCR. The overall *Sodalis* prevalence rate was 49.0%. The prevalence values varied significantly (*p* = 0.004) between localities; 70.8%, 54.7%, 38.5%, and 31.6%, respectively, in Peni, Bama, Bana, and Nasso (Table 2). None of the four *G. tachhnoides* harbored *S. glossinidius*. There was no association (*p* = 0.681) between the sex of the fly and the *Sodalis* infection.

**Figure 2:**
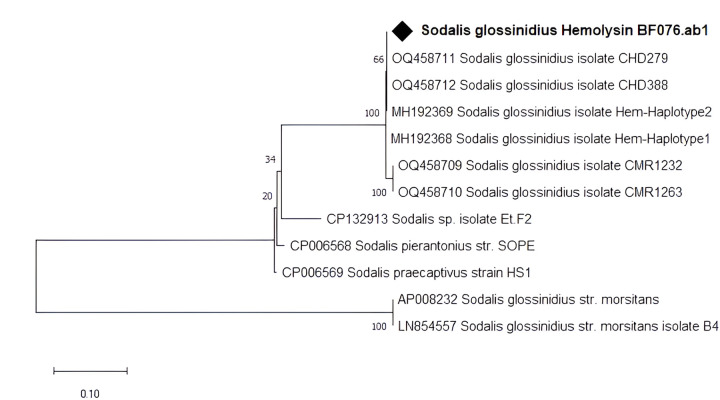
Phylogenetic analysis of *S. glossinidius* hemolysin partial gene sequence and its closed relatives. Our isolate is marked by a black diamond. The evolutionary history conducted in MEGA X [33] was inferred by using the Maximum Likelihood method and Kimura 2-parameter model [34]. The tree with the highest log likelihood (−2083.77) is shown. The percentage of trees in which the associated taxa clustered together is shown next to the branches. This analysis involved 12 nucleotide sequences.

The sequences of our amplicons had 100% similarity to *Sodalis’s Hemolysin* partial gene sequences (OQ458712.1, MH192369.1, LN854557.1) in the NCBI database. Phylogenetic relationships inferred together with reference sequences retrieved from GenBank showed that our amplicon clustered together with isolates from Chad (Fig. 2).

### Occurrence of Spiroplasma in in tsetse flies

**Figure 3:**
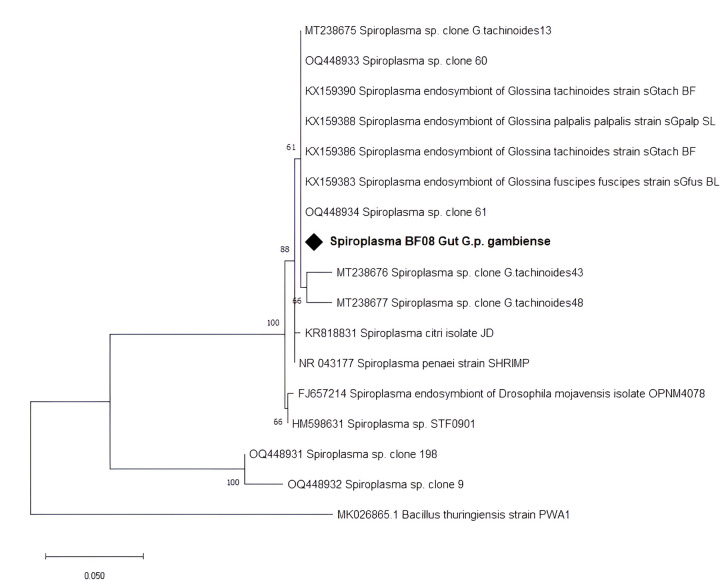
Phylogenetic analysis of *Spiroplasma*16S rRNA partial gene sequence and its closed relatives. Our isolate is marked by a black diamond. The evolutionary history conducted in MEGA X [33] was inferred by using the Maximum Likelihood method and Kimura 2-parameter model [34]. The tree with the highest log likelihood (−927.62) is shown. The percentage of trees in which the associated taxa clustered together is shown next to the branches. This analysis involved 17 nucleotide sequences. There were a total of 307 positions in the final dataset.

Screening for the presence of *Spiroplasma* was carried out using 16S rRNA-based PCR with wspecF/wspecR primers. *Spiroplasma* was detected in 193 (96.5%) of the samples, with a prevalence significantly lower (*p* < 0.0001) in Nasso (87.7%), while *Spiroplasma* was detected in 100% of samples from the three other collection sites (Table 2). All the four *G. tachhnoides* harbored *Spiroplasma*. There was no association (*p* = 0.802) between the sex of the fly and *Spiroplasma* infection. The sequence of our amplicons had > 98% similarity to *Spiroplasma* 16S rRNA partial gene sequences (KX159386.1, KX159388.1, OQ448933.1) in the NCBI database. The Maximum Likelihood phylogenetic tree (Fig. 3) revealed our amplicon clustered together with other *Spiroplasma* isolates from Burkina Faso and Cameroon.

### Occurrence of Wolbachia in tsetse flies

**Figure 4:**
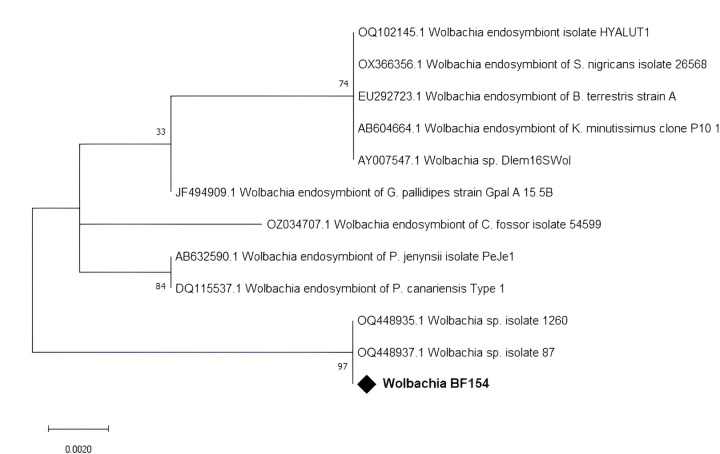
Phylogenetic analysis of *Wolbachia*16S rRNA partial gene sequence and its closed relatives. Our isolate is marked by a black diamond. The evolutionary history conducted in MEGA X [33], was inferred by using the Maximum Likelihood method and Kimura 2-parameter model [34]. The tree with the highest log likelihood (−519.94) is shown. The percentage of trees in which the associated taxa clustered together is shown next to the branches. This analysis involved 12 nucleotide sequences. There were a total of 332 positions in the final dataset.

From the 200 gut samples screened for the presence of *Wolbachia* using a 16S rRNA gene based PCR approach with wspecF/wspecR primers, 90 (45.0%) were confirmed to carry *Wolbachia* (Table 2). The highest *Wolbachia* prevalence in the flies analysed was found in Peni (70.8%), compared to that of Bama (43.4%), Bana (38.5%), and Nasso (38.6%). None of the four *G. tachhnoides* was infected by *Wolbachia*. There was no association (*p* = 0.226) between the sex of the fly and *Wolbachia* infection. The sequence of our amplicons had > 98% similarity to *Wolbachia* 16S rRNA gene sequences (OQ448937.1, OQ448935.1, OZ034998.1) in the NCBI database. Phylogenetic analysis with reference sequences retrieved from GenBank showed that our amplicon clustered together with isolates from Cameroon and Chad (Fig. 4).

### Coexistence of Sodalis, Spiroplasma, and Wolbachia

**Figure 5:**
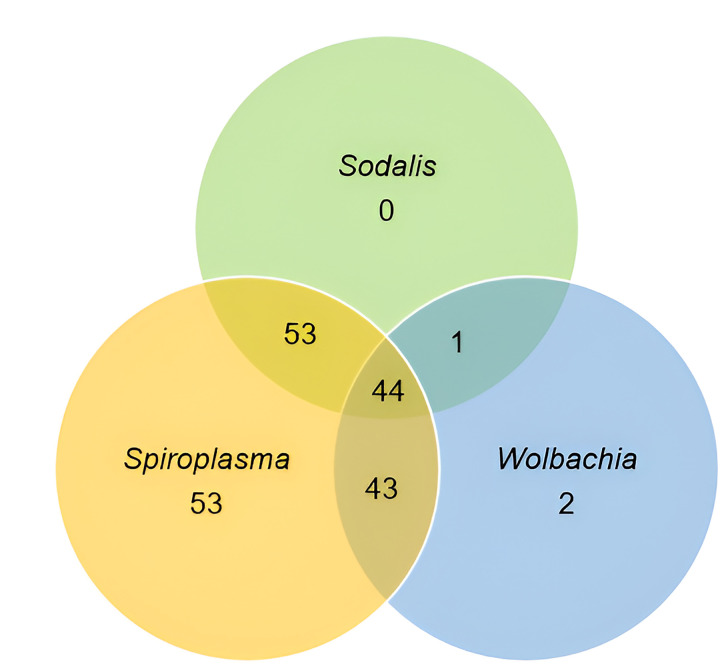
Coexistence of *Sodalis, Spiroplasma, and Wolbachia*. Endosymbionts co-existed in 141 tsetse flies. A total of 97 flies carried double infections: *Spiroplasma + Wolbachia* (43), *Sodalis + Wolbachia* (1), *Sodalis* + *Spiroplasma* (53), and 44 triple infection (*Sodalis + Spiroplasma + Wolbachia*).

A total of 196 (98.0%) flies were positive for at least one of the three symbionts (Fig. 5). Fifty-five (27.5%) were carrying single endosymbiont (*Spiroplasma* 26.5% and *Wolbachia* 1.0%), while 141 (70.5%) were carrying mixed infections with 97 (48.5%) carrying double infections (*Spiroplasma + Wolbachia* (21.5%), *Sodalis + Wolbachia* (0.5%), *Sodalis* + *Spiroplasma* (26.5%)), and 22.0% *Sodalis + Spiroplasma + Wolbachia*.

### Prevalence of trypanosomes in tsetse flies

Trypanosomes were present in some tsetse flies from all study locations, with an overall prevalence being 29.5% (59/200). In Bama, Bana, Nasso, and Peni, the trypanosome prevalence values were 39.6%, 23.1%, 8.8%, and 37.5%, respectively (Table 2). None of the captured *G. tachinoides* had trypanosomes.

**Figure 6:**
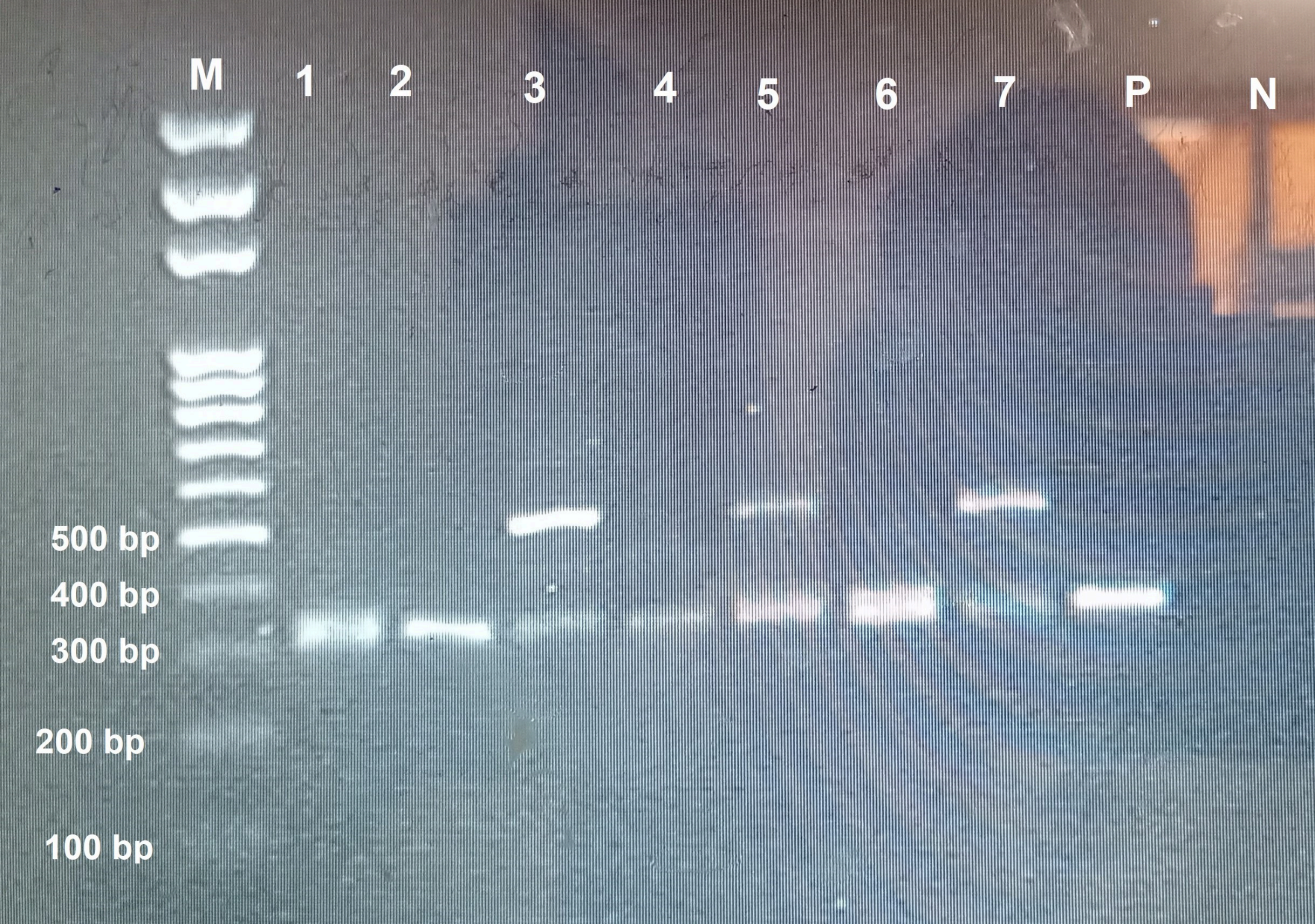
Agarose gel of selected ITS-1 amplicons. Lane M: Molecular Marker 100 bp; Lane 1: *T. grayi*; Lane 2: *T. grayi*; Lane 3: *T. grayi* and kinetoplastid; Lane 4: *T. grayi*; Lane 5: *T. grayi* and kinetoplastid; Lane 6: *T. grayi*; Lane 7: *T. grayi* and kinetoplastid; Lane P: Positive control; Lane N: Negative control.

*Trypanosoma grayi* was found in 59 samples (29.5%) and was the sole trypanosome species that was identified. In 22 *T. grayi*-positive samples, the agarose gel showed a second band at approximately 450–500 bp (Fig. 6) that we first identified as *T. brucei*.

**Figure 7:**
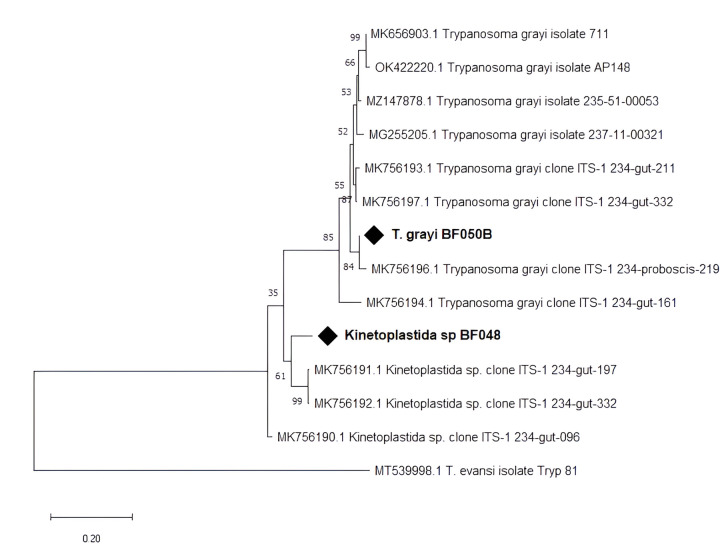
Phylogenetic analysis of *Trypanosoma*and kinetoplastidae ITS-1 gene sequences. Our isolate is marked by a black diamond. The evolutionary history conducted in MEGA X [33], was inferred by using the Maximum Likelihood method and Kimura 2-parameter model [34]. The tree with the highest log likelihood (−1054.41) is shown. The percentage of trees in which the associated taxa clustered together is shown next to the branches. A discrete Gamma distribution was used to model evolutionary rate differences among sites. This analysis involved 14 nucleotide sequences.

After sequencing and BLAST search, the result indicated that the sequences were not from *T. brucei*, although indicating a kinetoplastid origin. The sequences were 85.89% similar to *Kinetoplastida* sp. (MK756192.1). All 22 kinetoplastid bands were in a mixed infection with *T. grayi*. There was no association (*p* = 0.750) between the sex of the fly and trypanosome infection. The *T. grayi* sequence was closely related to *T. grayi* clone ITS-1–234 (MK756196, MK756198). It fell within the same branch with Nigerian *T. grayi* isolate (Fig. 7).

### Coexistence of symbionts and trypanosomes in tsetse flies

Of the 98 tsetse flies that were positive for *Sodalis*, 33 (33.7%) harbored *T. grayi* (Table 3). Of the 193 tsetse flies that were positive for *Spiroplasma, T. grayi* was present in 30.1% (58/193). Of the 90 tsetse that were positive for *Wolbachia*, the *T. grayi* was present in 22.2% (20/90). Similarly, of all trypanosome-infected flies, 55.9%, 98.3%, and 33.9% hosted *S. glossinidius, Spiroplasma* sp. and *Wolbachia*, respectively.

Analysis of the association between trypanosomes and endosymbiont infection in the trapped tsetse flies (Table 3) found no association between trypanosome infection and *Sodalis* and *Spiroplasma* (*p* > 0.05). However, there was a negative association with *Wolbachia* presence (*p* = 0.041).

The binary logistic regression (Table 4) found a 1.9 (95% CI 1.02–3.61) times likelihood of no trypanosome infection when *Wolbachia* is present.

## Discussion

The vector competence of tsetse flies for trypanosomes is highly variable and is suggested to be affected by various factors, among them is the presence of symbiotic bacteria. Here, we investigated the presence of three tsetse endosymbionts and their association with trypanosome infection in wild tsetse flies collected at four locations in Burkina Faso.

The entomological survey has revealed the presence of only two species of tsetse flies (*G. p. gambiense* and *G. tachinoides*) at the study locations. However, not far from our study area, in the South-West Burkina Faso, a previous study detected four tsetse species (*G. p. gambiense*, G*. tachinoides, G. m. submorsitans* and *G. medicorum*) [35]. The high frequency of *G. p. gambiense* compared to *G. tachinoides* suggests a better adaptation of *G. p. gambiense* to the environmental and climatic conditions of the area. The high density of tsetse in Bama and Nasso (10.7 and 7.3 respectively) compared to Bana and Peni (1.3 and 1.2, respectively) can be explained by the presence of a forest gallery and permanent water from Kou river, with diverse feeding sources (wild animals, reptiles, and livestock), which have created favourable microenvironment for tsetse flies.

Also, this study provides an overview of trypanosome species in the gut of tsetse from different localities around Bobo-Dioulasso, Burkina Faso. Using molecular tools (ITS1 amplification), we detected no *Trypanosoma* species diversity throughout the sampling sites. Only *T. grayi* was detected. Additionally, another non-identified kinetoplastid organism was found in 22 tsetse flies. The presence of *T. grayi* in these tsetse is not surprising since the area is densely inhabited by reptiles: crocodiles (*Crocodylus niloticus*) and monitor lizards (*Varanus niloticus*) [21]. Originally isolated from the crocodile, *T. grayi* has been considered a parasite of reptiles, corroborating many reports of this trypanosome in riverine species, in particular *G. palpalis*, feeding on reptiles [36, 37]. To the best of our knowledge, this is the first survey reporting the presence of *T. grayi* in tsetse flies from Burkina Faso. Meanwhile, there are recently consistent reports of this trypanosome species in Central Africa [26, 38–42]. *Trypanosoma grayi* has not been shown to be parasitic to humans or livestock, although one case was found in cattle in Cameroon [26] and few other cases in Nigerian cattle [42].

A 450–500 bp amplicon was amplified together with *T. grayi* in 22 samples. A similar observation was made by Weber et al. [42] in tsetse collected in several localities in Nigeria. They also similarly observed that the 500-bp amplicon of unidentified origin was always detected together with *T. grayi*. In another study where *T. grayi* were identified [26], there were also unexpected and non-identified amplicons that were later sequenced and identified as deriving from Bodonidae kinetoplastid DNA. Further investigations are needed to identify these unknown PCR amplicons. The organism could be another strain of *T. grayi*.

The trypanosome prevalence found in this study (29.5%) is higher than 16.8%, 10.5%, and 18.1%, respectively, found in *G. tachinoides*, *G. p. gambiense* and *G. m. submorsitans* and lower than 39.6% found in *G. medicorum* in a previous study in other localities of Burkina Faso [20]. However, different trypanosome species (*T. congolense*, and *T. vivax*) were identified. Surprisingly, only one trypanosome species (*T. grayi*) was detected in this study, while previous studies on domestic animals in other localities in Burkina Faso found other species including *T. congolense, T. brucei*, and *T. vivax* [43, 44].

The overall symbiont occurence rate was relatively high, with 98% of flies that harbored at least one of the three endosymbionts. The *S. glossinidius* occurence rate of 49.0% obtained in the present study is higher than 0.5%, 2.0%, and 9.0% previously reported in Burkina Faso, Cameroon, and Chad respectively [19, 27, 45]. It is important to point out that, in the present study, *S. glossinidius* was identified using Hem primers (targeting the nuclear hemolysin gene), which are reported to provide a more reliable assessment of prevalence than the pSG2 primers (targeting the plasmid2) used in previous studies [46]. However, this prevalence is also lower than 54.9% and 93.7% reported for *G. p. palpalis* in Cameroon [47] and for *G. brevipalpis* in Zambia, respectively [48]. Moreover, the present work indicates a lack of association between *Sodalis* and trypanosome infection. It has been the same for other studies [49, 50]. However, several studies reported a potential positive association between *Sodalis* and trypanosome infections, leading to the hypothesis that *Sodalis* might favour the establishment of Trypanosome infections in the tsetse midgut [19, 45, 47, 51]. The hypothesis is based on lectin-inhibitory activity, in which chitinase from *S. glossinidius* breaks down chitin and produces N-acetyl-D-glucosamine, which inhibits lectin function in the flies [52].

Comparing the prevalence of *Wolbachia* between wild populations of different tsetse species of various regions, our prevalence of 45.0% is higher than 9.7% and 14.5% reported for *G. p. palpalis* in Cameroon [27] and *G. m. submorsitans* in Chad [45], but lower than 80.5% and 78.9% reported in Zambia for *G. m. morsitans* and *G. pallidipes*, respectively [53]. The variation in these results can be due to the difference in tsetse species, the sensitivity of molecular markers, the analysed tissue, the sample collection period, and the geographical and climatic conditions. Some previous studies did not find involvement of *Wolbachia* in tsetse vector competence [45, 49]. However, our study found a negative association between trypanosome infection and the presence of *Wolbachia*. Other recent investigations found a negative correlation between *Wolbachia* and the presence of trypanosomes [54, 55], although the prevention mechanism is not yet understood. Similar observations have already been described for other vector-borne diseases. *Wolbachia* infections were found to limit mosquito-transmitted pathogens including, Dengue virus, Chikungunya virus, Plasmodium parasites, yellow fever virus, and Zika virus [56–58]. The negative association of a specific strain of *Wolbachia* with trypanosome infections, could open new perspectives for vector control and the development of paratransgenic approaches where trypanosome establishment could be prevented in the gut.

The *Spiroplasma* general infection rate of 96.5% obtained in the present study is higher than 44.5% reported in Uganda for *G. fuscipes* (*f*.) *fuscipes* and 17.17% reported in Burkina Faso for *G. tachinoides* [12, 59]. The high variation of prevalence across sampling sites in Uganda’s study was correlated with the geographic origin and the season of collection of flies [12]. In this study, there was no association between *Spiroplasma* and trypanosome infection. However, in *G. f. fuscipes*, it was found that trypanosomes were less likely to establish an infection in individuals that carried *Spiroplasma* infections [12], suggesting that *Spiroplasma* infections may have an important effect on fly’s resistance to infection with trypanosomes. *Spiroplasma* was found to induce reproductive abnormalities, including changes in sex–biased gene expression, adepletion in the availability of metabolically critical lipids in pregnant females that results in delayed larval development, and compromised sperm fitness [60]. *Spiroplasma* could therefore be exploited in tsetse population reduction approach.

The discrepancy between the results on the association between trypanosome infection and symbiotic bacteria could be due to the difference in tsetse species or symbiont strains. The molecular characterisation of these endosymbionts could provide more information on their effect on tsetse vector competence.

## Conclusion

*Trypanosoma grayi* was the only trypanosome species found in this study. This is the first report of the presence of *T. grayi* in Bobo-Dioulasso. The three endosymbionts, *Sodalis, Spiroplasma* and *Wolbachia* were detected in the four collection sites. While *S. glossinidius* and *Spiroplasma* have no effect on trypanosome infections, *Wolbachia* has a negative association with trypanosome infection. More investigations are required to better understand interactions between symbionts and trypanosomes in tsetse. Data could help in the development of a paratransgenesis strategy to prevent trypanosome transmission.

## Figures and Tables

**Table 1:** Relative abundance of tsetse flies per collection site

Localities	No. flies Caught	No. traps Used	No. trapping days	Apparent Density	No. flies dissected	No. flies analysed
**Bama**	320 (74.4%)	10	3	10.7	154	104
**Bana**	13 (3.0%)	5	2	1.3	13	13
**Nasso**	73 (17.0%)	5	2	7.3	59	59
**Peni**	24 (5.6%)	10	2	1.2	24	24
**Total**	**430**	**30**	**4**	**3.5**	**250**	**200**

**No.**= number of; **Apparent Density** in flies per trap per day (F/T/D)

**Table 2:** Symbionts and trypanosomes distribution per location

Localities	Tsetse species	*Sodalis*	*Spimplasma*	*Wolbachia*	*Trypanosoma* sp.
**Bama**	*G.p.g.*	58/106 (54.7%)	106/106 (100%)	46/107 (43.4%)	42/106 (39.6%)
**Bana**	*G.p.g.*	5/13 (38.5%)	13/13 (100%)	5/13 (38.5%)	3/13 (23.1%)
**Nasso**	*G.p.g.*	18/53 (33.9%)	46/53 (86.8%)	22/53 (41.5%)	5/53 (9.4%)
**Nasso**	*G.t.*	0/4 (0.0%)	4/4 (100%)	0/4 (0.0%)	0/4 (0.0%)
**Peni**	*G.p.g.*	17/24 (70 8%)	24/24 (100%)	17/24 (70.8%)	9/24 (37.5%)
**Total**		**98/200 (49.0%)**	**193/200 (96.5%)**	**90/200 (45.0%)**	**59/200 (29.5%)**

**G.p.g.** = *G.p. gambiense* ; **G.t.** = *G. tachinoides*

**Table 3:** Symbionts-trypanosomes association in tsetse flies

Trypanosome infection	*Sodalis*		*Spimplasma*		*Wolbachia*	
	Negative	Positive	Negative	Positive	Negative	Positive
Absent	76	65	6	135	71	70
Present	26	33	1	58	39	20
**p (Chi-2)**	0.205		0.369		0.041	

**Table 4:** Relationship between the presence of endosymbionts (*Sodalis, Spiroplasma and Wolbachia*) and the trypanosome infections in tsetse flies

Symbionts	No	S+	T+	S + T+	S + T−	S−T+	S−T−	*p*	OR	95% CI
**Sodalis**	200	98	59	33	65	26	76	0.206	0.674	0.366–1.242
**Spiroplasma**	200	193	59	58	135	1	6	0.386	0.388	0.046–3.295
**Wolbachia**	200	90	59	20	70	39	71	**0.043**	1.923	1.022–3.617

**No**: Number of analyzed samples **S+**: Symbiont positive; **S−**: Symbiont negative;

**T+**: Trypanosome positive; **T−**: Trypanosome negative; **OR**: Odds ratio

## Data Availability

The datasets generated and analysed during this study are included in this publication. The 16S rRNA, the ITS-1, and the hemolysin partial gene sequences generated have been deposited in the NCBI database (GenBank) under accession numbers PQ046014, PQ046015, PQ067144, PQ045884, and PQ045885.
